# Fabrication of Te and Te-Au Nanowires-Based Carbon Fiber Fabrics for Antibacterial Applications

**DOI:** 10.3390/ijerph13020202

**Published:** 2016-02-06

**Authors:** Ting-Mao Chou, Yi-Yun Ke, Yu-Hsiang Tsao, Ying-Chun Li, Zong-Hong Lin

**Affiliations:** Institute of Biomedical Engineering, National Tsing Hua University, Hsinchu 30013, Taiwan; tommy0703@outlook.com (T.-M.C.); orzhappy@hotmail.com (Y.-Y.K.); chrnoblue@gmail.com (Y.-H.T.); kkccll1188@gmail.com (Y.-C.L.)

**Keywords:** tellurium, gold, antibacterial, nanowire, carbon fiber, fabric

## Abstract

Pathogenic bacteria that give rise to diseases every year remain a major health concern. In recent years, tellurium-based nanomaterials have been approved as new and efficient antibacterial agents. In this paper, we developed the approach to directly grow tellurium nanowires (Te NWs) onto commercial carbon fiber fabrics and demonstrated their antibacterial activity. Those Te NWs can serve as templates and reducing agents for gold nanoparticles (Au NPs) to deposit. Three different Te-Au NWs with varied concentration of Au NPs were synthesized and showed superior antibacterial activity and biocompability. These results indicate that the as-prepared carbon fiber fabrics with Te and Te-Au NWs can become antimicrobial clothing products in the near future.

## 1. Introduction

Semiconductor nanorods (NRs) and nanowires (NWs) are interesting nanomaterials (NMs) and have been applied in sensor and energy applications [1,2,3,4]. Tellurium (Te) is a typical p-type semiconductor with a bandgap energy of 0.35 eV and has already been recognized as an interesting material for fabricating nanodevices. For examples, Te NWs have shown great potential in the applications of nanogenerators [5,6,7], supercapacitors [8], lithium batteries [9], and biosensors [10,11]. 

In recent years, scientists have started to study the antibacterial and biocompatible properties of Te NMs. Four differently shaped Te NMs (nanocubes, nanorices, nanopencils, and nanowires) have been synthesized and investigated for the antibacterial mechanism against *Escherichia coli* (*E. coli*) [12]. Based on the results, tellurite (TeO_3_^2‒^) ions released from the four Te NMs account for the antibacterial activity. Compared to silver nanoparticles (Ag NPs) that are commonly used as antibacterial reagents, Te NMs have higher antibacterial activity and lower toxicity. Then Au-Te and Au-Ag_2_Te NMs were prepared and showed superior antibacterial activity in the presence of light irradiation [13,14]. These NMs have a relatively large surface area and can generate reactive oxygen species (ROS) when exposed to solar light. The capabilities of the controlled release of active ions (TeO_3_^2‒^ and Ag^+^) and the generation of ROS make these NMs inhibit the growth of *E. coli* and *Staphylococcus aureus* (*S. aureus*) either in the dark or in the presence of light irradiation. In addition, *in-vitro* cytotoxicity assays have revealed their low toxicity in human cell lines and red blood cells [14]. 

Regarding the application of Te-based NMs as antibacterial agents in daily life, it is necessary to directly prepare functional substrates with Te NMs. In this paper, we developed an approach to grow Te NWs onto commercial carbon fiber fabrics. In the presence of Te NWs, the fabrics resulted in the inhibition of bacterial growth by slowly releasing active tellurite ions. These Te NWs also served as templates and reducing agents for the deposition of Au NPs. The size of Au NPs can be controlled by varying the Au^3+^ concentration. Compared to Te NWs, Te-Au NWs provided stronger antibacterial activity due to the generation of ROS. 

## 2. Experimental Section

### 2.1. Chemicals

Sodium dodecyl sulfate (SDS, ≥98.5%) hexadecyltrimethylammonium bromide (CTAB, 99%), and hydrogen tetrachloroaurate (HAuCl_4_, ACS reagent, ≥49.0% Au basis) were obtained from Sigma-Aldrich. Tellurium dioxide (TeO_2_, 99.9%) and hydrazine monohydrate (80%) were purchased from Showa. The BacLight Bacterial Viability and Counting Kit were acquired from Invitrogen. Ultrapure water from a Milli-Q ultrapure system (18.2 MΩ cm) was used throughout this study.

### 2.2. Growth of Te and Te-Au Nanowire Arrays on Carbon Fiber Fabrics

Before the growth of Te NWs, the carbon fiber fabrics were cleaned with isopropanol and water. Then the carbon fiber fabrics were put in the hydrazine solution containing 10 mM SDS and 1 mM TeO_2_. After a growth time of 2 h, the carbon fiber fabrics with Te NWs were obtained. To further grow Au NPs on the Te NWs, the carbon fiber fabrics with Te NWs were washed with water twice and then put in the HAuCl_4_ solution. The size and density of Au NPs on Te NWs can be controlled by reacting with different concentrations of HAuCl_4_ solution. The reaction time was fixed at 30 min. Finally, the prepared carbon fiber fabrics with Te and Te-Au NWs were dried in air at room temperature prior to characterization and antibacterial test.

### 2.3. Characterization

A UV/Vis spectrophotometer (V730, Jasco) was used to measure the absorption spectra of Te and Te-Au NWs. JEOL JEM-1200 EX II transmission electron microscopes (TEM) and JSM-7600F field emission scanning electron microscope (FESEM) were used to measure the size and shape of the prepared Te and Te-Au NWs. An energy dispersive X-ray system (Inca Energy X-Max, Oxford, UK) was used to determine the composition of the prepared NWs. Inductively coupled plasma-mass spectrometry measurements were conducted on an Agilent 7700e instrument to determine the concentration of Te-Au NWs and the released tellurite ions. A Hitachi F-7000 fluorescence spectrophotometer was used to record the fluorescence of the bacteria stained with SYTO 9 and propidium iodide (PI). 

### 2.4. Antibacterial and Cytotoxicity Tests 

*E. coli* DH5α and *S. aureus* cells were grown in LB media. A single colony from LB agar plates was inoculated in LB medium (10 mL) and the culture was grown overnight until the value of A_670_ reached 0.06 (*E. coli*) and (*S. aureus*). A portion of each cell sample was centrifuged (4000 rpm, 10 min) and washed twice with 0.85% sodium chloride to remove matrices. Cells diluted to 2.0 × 10^8^ bacteria/mL (*E. coli*) and 2.0 × 10^7^ bacteria/mL (*S. aureus*) were mixed separately with the as-prepared carbon fiber fabrics in 0.85% sodium chloride (25 °C, 10 and 30 min). The viability assay was conducted using SYTO 9 and PI stains. We collected cells treated with the as-prepared carbon fiber fabrics. The suspension (100 μL) was dispensed in a quartz cell and then 100 μL of the SYTO 9 and PI stains was added to the solution. The mixture was incubated for 15 min at room temperature. Fluorescence intensities of SYTO 9 (excitation: 475 nm, emission: 530 nm) and PI (excitation: 475 nm, emission: 640 nm) were recorded separately. The green/red fluorescence ratio was used to calculate the percentage of viable bacteria. Regarding the *in-vitro* cytotoxicity test, MCF-10A mammary epithelial cells were cultured in RPMI-1640 supplemented with FBS (10%), ampicillin (1%) in 5% CO_2_ at 37 °C. The cell number was determined by the trypan blue exclusion method. Cell viability was determined using an MTT assay. MCF-10A (1.5 × 10^5^ cells/well) in a culture medium containing 5% CO_2_, were incubated with the as-prepared carbon fibers for 24 h at 37 °C. Then the cells were carefully rinsed thrice with phosphate buffered saline and treated with MTT reagent. Relative viable cell levels were determined by the MTT assay according to the ELISA Reader. 

## 3. Results and Discussion

### 3.1. Preparation and Antibacterial Mechanism of the As-Prepared Carbon Fiber Fabrics 

**Figure 1:**
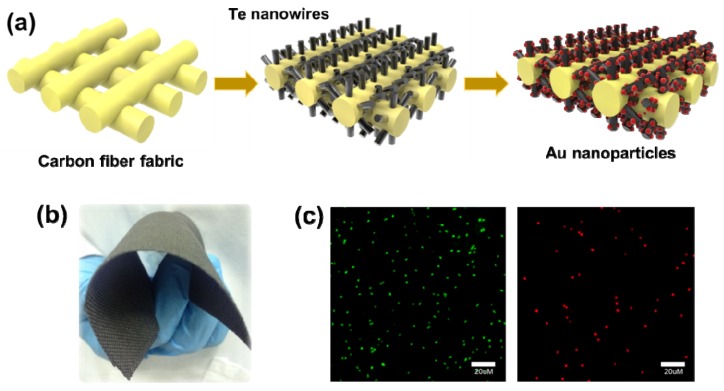
(**a**) Illustration of the growth of Te and Te-Au NWs on carbon fiber fabrics; (**b**) Photograph of the as-prepared carbon fiber fabrics with Te-Au NWs; (**c**) Fluorescence images of live (green) and dead (red) bacteria.

Figure 1 illustrates the preparation and antibacterial mechanism of the carbon fiber fabrics with Te and Te-Au NWs. The synthesis of Te NWs followed a green chemistry approach [15]. Before the synthesis, the commercial carbon fiber fabrics were ultrasonically cleaned with acetone, ethanol, and water, respectively. By using concentrated N_2_H_4_ solution as the reducing agent, the growth of Te NWs from TeO_2_ powder was achieved at ambient temperature (25 °C, 2 h). From the point of green chemistry, this approach is favorable over others in terms of energy-saving. Through a redox reaction between Au^3+^ ions and the Te NWs, Au atoms were deposited to form Au NPs on the Te NWs, while tellurite ions were formed and released into the solution [16]. In a typical synthesis, the carbon fiber fabrics with Te NWs are immersed in a solution containing 10 mM CTAB and 1 mM HAuCl_4_ for 30 min. By controlling the concentration of HAuCl_4_ solution, different sizes of Au NPs can be formed on the Te NWs. Finally, the carbon fiber fabrics with Te and Te-Au NWs were washed by water and dried at ambient temperature for the characterization and antibacterial study. The photograph shown in Figure 1b is the prepared carbon fiber fabrics with Te-Au NWs, which clearly shows the potential of becoming clothing products in the near future. The antibacterial activity of Te and Te-Au NWs is due to their natural oxidation in aqueous solution [17], which results in the formation of tellurite ions. Tellurite ions are toxic to most bacteria, particularly Gram-negative ones [18,19,20,21]. The antibacterial activity of the carbon fiber fabrics with Te and Te-Au NWs against *E. coli* and *S. aureus* can be evaluated by using SYTO 9 and PI dyes to stain the cells. The mechanism is based on the fact that SYTO 9 with green fluorescence can enter all cells, while PI with red fluorescence is excluded from cells with intact cytoplasmic membranes [22]. Therefore, with an appropriate mixture of the SYTO 9 and PI stains, bacteria with intact cell membranes stain fluorescent green, whereas bacteria with damaged membranes stain fluorescent red. The green and red cells shown in Figure 1c correspond to live and dead *E. coli*, respectively. The viable cells in bacteria solution are used as an indicator of antibacterial activity.

### 3.2. Characterization of the As-Prepared Carbon Fiber Fabrics with Te and Te-Au NWs

**Figure 2:**
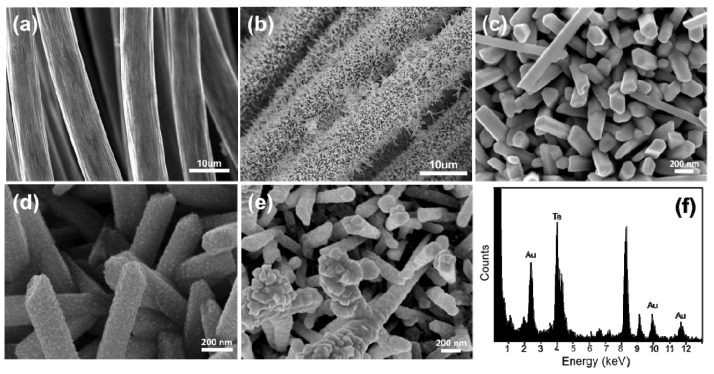
(**a**–**c**) SEM images of the carbon fiber fabrics before (**a**) and after (**b**,**c**) the growth of Te NWs; (**d**,**e**) SEM images of the Au-Te NW-based carbon fiber fabrics when using Te NW-based carbon fiber fabrics to react with 0.5-mM (**d**) and 2-mM (**e**) HAuCl_4_; (**f**) EDX spectrum of the Au-Te NW-based carbon fiber fabrics.

The SEM images in Figure 2a,b represent the commercial carbon fiber fabrics before and after the growth of Te NWs. Before the growth of Te NWs, the surface of carbon fiber fabrics was clean. After the growth of Te NWs, nanowire arrays were uniformly formed on the carbon fiber fabrics. Te NWs with a diameter of 155 nm and a length of 1.3 µm were synthesized through the redox reaction between N_2_H_4_ and TeO_3_^2‒^ ions (Figure 2c). The length of Te NWs can be controlled by either adjusting the concentration of N_2_H_4_ or TeO_2_. After washing with water to remove unbinding Te NWs, the as-prepared carbon fiber fabrics were immersed in HAuCl_4_ solution to grow Au NPs. The growth of Au NPs is based on another spontaneous redox reaction between Au^3+^ and Te NWs:

4Au^3+^ + 3Te + 6H_2_O   4Au + 3TeO_2_ + 12H^+^ (1)

The growth of Au NPs on Te NWs can be controlled by varying the solution pH or Au^3+^ concentration [16]. The control of solution pH can tune the redox reaction rate of Au^3+^ and Te NWs, which consequently affects the rate of formation of the Au seeds. At higher pH (pH > 7), Au NPs would deposit at various positions along Te NWs to form Au-Te nanopeapods. However, Au NPs would selectively grow on the ends of Te NWs at a lower pH (pH < 5). In this paper, we controlled the redox reaction of Au^3+^ and Te NWs at a higher pH (pH = 9). When the as-prepared carbon fiber fabrics with Te NWs were immersed in a solution containing 10 mM CTAB and 0.5 mM HAuCl_4_ for 30 min, Au NPs with a size of 24 nm were formed on the surface of Te NWs (Figure 2d). When the concentration of HAuCl_4_ was changed to 2 mM, the size of Au NPs on Te NWs increased to 76 nm (Figure 2e). Additionally, the Au NPs almost covered all the surface of Te NWs. We conducted EDX and ICP-MS measurements to confirm the compositions of NMs on the as-prepared carbon fiber fabrics. The representative EDX data clearly shows that the composition of NPs on the surface of Te NWs is Au (Figure 2f). The ICP-MS results are in good agreement with the EDX data. When using a HAuCl_4_ concentration of 0, 0.5, 1, and 2 mM, we obtained Te-Au_n_ NWs with values of n of 0, 0.2, 0.5, and 0.8, respectively. The zeta potentials of the as-prepared Te and Te-Au NWs were also measured. The Te and Te-Au NWs were removed from the carbon fiber fabrics through a sonication process and dispersed in deionized water. Before the growth of Au NPs, Te NWs were stabilized by SDS and the measured zeta potential was −21.2 mV. During the growth of Au NPs, the stabilizer has been changed to CTAB. Additionally, after the growth of Au NPs, the measured zeta potential was +13.2, +15.4, +17.8 mV for Te-Au_0.2_ NWs, Te-Au_0.5_ NWs, and Te-Au_0.8_ NWs.

### 3.3. Electronic States and Optical Properties of the Te and Te-Au NWs

**Figure 3:**
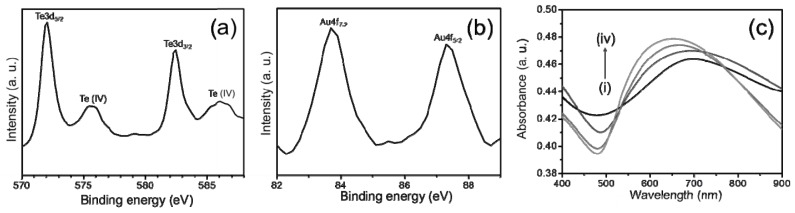
(**a,b**) XPS spectra of the Te-Au NWs on carbon fiber fabrics; (**c**) UV/Vis absorption spectra of the Te NW-based carbon fiber fabrics reacting with 0 (i); 0.5 mM (ii); 1 mM (iii); and 2 mM (iv) HAuCl_4_.

The electronic states of the Te-Au NWs on carbon fiber fabrics were further confirmed by X-ray photoelectron spectroscopy (XPS), which revealed the existence of Au and Te elements in the NWs. The peaks at 572.5 eV and 582.4 eV correspond to Te 3d_5/2_ and Te 3d_3/2_ (Figure 3a), respectively. Additionally, the peaks at 83.5 eV and 87.2 eV correspond to Au 4f_7/2_ and Au 4f_5/2_ (Figure 3b), respectively. Along with two major tellurium peaks, two minor peaks are observed at 576.1 eV and 586.4 eV, which can be attributed to Te (IV) oxide. The XPS data are similar to those reported previously. The optical properties of the Te and Te-Au NWs were determined by UV/Vis absorption spectra (Figure 3c). A distinct absorption band of the Te NWs occurs at around 690 nm (curve i), which is due to the electron transition from the valence band to the conduction band [23,24]. Curves ii‒iv of the three as-prepared Te-Au NWs exhibited increased surface plasma resonance (SPR) absorbance at 570 nm upon increasing the amount of Au in the Te NWs. The characteristic absorbance at 690 nm of the Te NWs gradually decreased upon progressing from the Te NWs to Te-Au NWs with more gold deposition. In addition, their maximum absorption wavelengths underwent blue shifts. These features resulted mainly from the increased Au content and decreased Te content. The absorption profile of Au NPs is similar to the previous reports about Te-Au NMs [15,16]. 

### 3.4. Antibacterial Activity of the As-Prepared Carbon Fiber Fabrics with Te and Te-Au NWs

**Figure 4:**
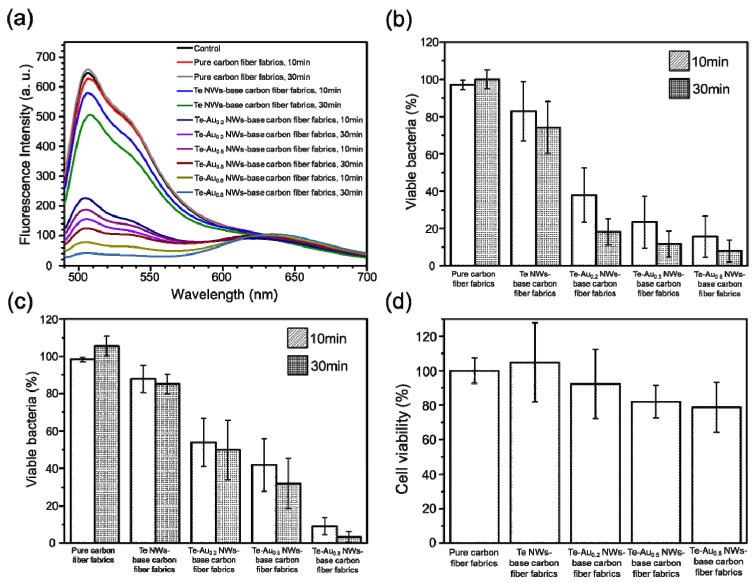
The antibacterial activity and cytotoxicity of carbon fiber fabrics with Te and Te-Au NWs. (**a**) Fluorescence spectra of the *E. coli* solutions (2.0 × 10^8^ bacteria/mL) stained with SYTO 9 and PI. The as-prepared carbon fiber fabrics were immersed in the *E. coli* solutions for 10 and 30 min. The emission ratio of 530 nm/640 nm was used as an indicator to evaluate the antibacterial activity; Viable (**b**) *E. coli* and (**c**) *S. aureus* cells were calculated by the emission ratio of 530 nm/640 nm; (**d**) Cell viability of MCF-10A cells incubated with the Te and Te-Au NW-based carbon fiber fabrics for 24 h.

The percent viability of *E. coli* and *S. aureus* with/without the treatment of the as-prepared carbon fiber fabrics was obtained from the fluorescence measurement (Figure 4). The viable bacteria were used as an indicator to evaluate the antibacterial activity of as-prepared carbon fiber fabrics with Te and Te-Au NWs. The fluorescence approach allowed counting a large number of bacterial cells at the same time, which provided more reliable data than those obtained by counting the bacteria under the microscope. Firstly, the as-prepared carbon fiber fabrics with Te and Te-Au NWs were immersed in the *E. coli* solution (2.0 × 10^8^ bacteria/mL) for 10 and 30 min. As a control, the antibacterial activity of pure carbon fiber fabrics was also examined. Figure 4a shows the fluorescence spectra of the *E. coli* suspensions stained by SYTO 9 and PI. The emission ratio of green light (530 nm)/red light (640 nm) was used as an indicator to calculate the viable bacteria (Figure 4b). After the treatment with pure carbon fiber fabrics for 30 min, 98% of *E. coli* cells remained alive; thus, the pure carbon fiber fabrics showed no antibacterial activity. The viable *E. coli* incubated with the Te NW-based carbon fiber fabrics was 82% after 10 min and further decreased to 73% after 30 min. Compared to Te NW-based carbon fiber fabrics, Te-Au NW-based carbon fiber fabrics show better performance toward the inhibition of *E. coli* cells. Only 40% of *E. coli* cells remained alive after the treatment with Te-Au_0.2_ NW-based carbon fiber fabrics for 10 min, and around 20% were alive after the treatment for 30 min. This is because the presence of Au NPs on semiconductor NMs would help to generate ROS [13,16,25,26]. The antibacterial activity of the as-prepared carbon fiber fabrics continued to increase as more Au content deposited on the Te NWs. Te-Au_0.8_ NW-based carbon fiber fabrics provided the best antibacterial activity among the three Te-Au NW-based carbon fiber fabrics. More than 90% of the dead *E. coli* cells were observed after the treatment with Te-Au_0.8_ NW-based carbon fiber fabrics for 30 min. We also compared the antibacterial activity of the Te and Te-Au NW-based carbon fiber fabrics against *S. aureus* (2.0 × 10^7^ bacteria/mL). The antibacterial activity of the as-prepared carbon fiber fabrics against *S. aureus* is shown in Figure 4c. The decreasing order of the viability of *S. aureus* was pure carbon fiber fabrics > Te NW-based carbon fiber fabrics > Te-Au_0.__2_ NW-based carbon fiber fabrics > Te-Au_0.__5_ NW-based carbon fiber fabrics > Te-Au_0.8_ NW-based carbon fiber fabrics. After 30 min, the viability values were 103%, 87%, 51%, 36%, and 4% in the presence of pure carbon fiber fabrics, Te NW-based carbon fiber fabrics, Te-Au_0.__2_ NW-based carbon fiber fabrics, Te-Au_0.__5_ NW-based carbon fiber fabrics, and Te-Au_0.8_ NW-based carbon fiber fabrics, respectively. All these results have indicated that the as-prepared Te and Te-Au NW-based carbon fiber fabrics hold great practical potential as efficient antibacterial agents. Finally, the cytotoxicity of the as-prepared carbon fiber fabrics toward mammalian cells was evaluated using an MTT assay (Figure 4d). After a 24 h incubation of MCF-10A mammary epithelial cells with the as-prepared carbon fiber fabrics, we found that the Te-Au_0.8_ NW-based carbon fiber fabrics had little influence on cells (around 18%). The as-prepared carbon fiber fabrics therefore demonstrated biocompatibility toward mammalian cells. However, further studies using animal models should be conducted to ensure the application of being antimicrobial clothing products.

**Figure 5:**
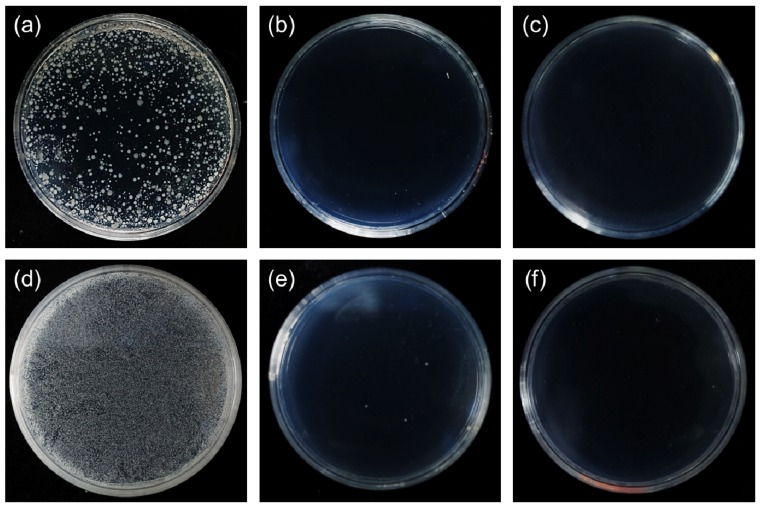
(**a**–**c**) The antibacterial activity against *E. Coli* by pure carbon fiber fabrics (**a**); Te NW-based carbon fiber fabrics (**b**); and Te-Au_0.8_ NW-based carbon fiber fabrics (**c**); (**d**–**f**) The antibacterial activity against *S. aureus* by pure carbon fiber fabrics (**d**); Te NW-based carbon fiber fabrics (**e**); and Te-Au_0.8_ NW-based carbon fiber fabrics (**f**). All the results were obtained based on AATCC 100 test method.

We also used the AATCC 100 test method to evaluate the antibacterial activity of the as-prepared carbon fiber fabrics with Te and Te-Au NWs. It determines both bacteriostatic activity (inhibition of multiplication) and bactericidal activity (killing of bacteria) of the NMs on fabrics. The AATCC antimicrobial test is more stringent. During the incubation period, bacteria are provided continuous nutrition and consequently will multiply unless fabrics provide significant antibacterial activity to reduce the number of test bacteria. Figure 5 shows the AATCC 100 test results toward *E. coli* and *S. aureus*. Microbial suspensions with 0.5 mL were loaded to 2.0 cm × 2.5 cm carbon fibers swatches. After a contact time of 24 h at 37 °C, the samples were vortexed with 1.5 mL of 0.85% NaCl solution. Serial dilutions of the quenched samples were made and then plated on nutrient agar. The plates were incubated at 37 °C for another 24 h. It can be observed that there is nearly 100% reduction of the number of colonies in both the samples treated by the as-prepared carbon fiber fabrics with Te and Te-Au_0.8_ NWs.

## 4. Conclusions

In summary, we have developed an approach to prepare Te and Te-Au NWs on commercial carbon fiber fabrics. The length of Te NWs can be controlled by either adjusting the concentration of reducing agent N_2_H_4_ or precursor TeO_2_. Through the spontaneous redox reaction between Au^3+^ and Te NWs, Au NPs can selectively grow on the Te NWs. Carbon fiber fabrics with different ratios of Te and Au were obtained by simply varying the Au^3+^ concentration. Comparing to Te NW-based carbon fiber fabrics, Te-Au NW-based carbon fiber fabrics show better performance toward the inhibition of *E. coli* and *S. aureus* cells. Less than 10% of the bacteria remained alive after the treatment with Te-Au_0.8_ NW-based carbon fiber fabrics for 30 min. We believe that the approach can be expanded to grow Te and Te-Au NWs on other kinds of fabrics. These results indicate that the fabrics with Te and Te-Au NWs can become antimicrobial clothing products in the near future.
